# Buffalo Medical and Surgical Association

**Published:** 1882-07

**Authors:** 


### Buffalo Medical and Surgical Association.

*Regular Meeting, Monday Evening, June 5, 1882.*

President, Dr. A. R. Davidson, in the chair. Dr. C. M. Daniels, Secretary.

Present—Drs. Cronyn, Rochester, Fowler, Storck, Howe, Harvey, C. A. Ring, Burkhardt, Runner, Moody, Bartow, O'Brien, Hartwig, Stockton, Hopkins, Brecht, Mynter, Bartlett, Van Peyma, McNeil, and by invitation Drs. McBeth, Frederick, Frank and Fell.

The minutes of the last meeting were read and approved.

*Essay*—By Dr. Lucien Howe, subject: "The Pupil in Disease." A full report of this paper appears in the June number of THE BUFFALO MEDICAL AND SURGICAL JOURNAL.

*Discussion*—Dr. Cronyn thought the essayist had succeeded in giving the members of the society something to think about, and considered the subject one of great importance well taken, and said it was now being considered extensively by several European physicians. He thought the conditions were as demonstrated, although the root of the difficulty may not yet be fully appreciated. Practically it was a matter of almost daily experience and worthy of a great deal of thought.

Dr. Fell remarked that he was very much interested in the paper, and felt like following up the course pursued.

Dr. Howe further spoke of the effect of chloroform upon the pupil, that it contracts and remains so, when it is gradually administered, but dilatation may be obtained by suddenly slapping the patient. When patient is fully anæsthetized a sudden dilatation indicates serious consequences.

Dr. Rochester said he had often noticed that chloroform contracts and ether dilates the pupil, and asked that Dr. Howe further experiment and report.

Dr. Howe thought dyspnœa was the cause of dilatation with ether.

*Voluntary Communication*—Dr. Hartwig presented specimens of fungoid growths removed from the knee-joint, and related manner of removal by scraping, &c. He recommended the use of iodoform in surgical dressings. The specimens were given to Dr. Fell, with the request that he examine them microscopically and report.

Dr. Mynter remarked that opening the knee-joint was a dangerous proceeding.

*Prevailing Diseases*—Dr. Rochester reported measles, roseola and a little scarlet fever, and gave an interesting history of several cases of diphtheria, where the mortality was evidently greatly increased by sewer gas, and took the ground that although sewer gas in itself may not propagate the disease, yet, where the disease is present, it seems to increase the fatality.

Dr. Cronyn thought the prevailing diseases were about the same as during May, and said he agreed with Dr. Rochester regarding the non-infecting power of sewer gas, although it doubtless assisted materially in advancing disease.

Dr. Hopkins had heard and read a good many extraordinary opinions about sewer gas, and asked the President or some other member of the society to enlighten them upon the subject by information as to what sewer gas is.

Dr. Davidson said that a good deal of alarm was caused by sensational statements of plumbers and others concerning the noxious and deadly nature of sewer gas. In fact there is no such thing as sewer gas. That so-called, being simply air more or less contaminated with carbonic acid and sulphuretted hydrogen. These gases are readily detected by testing with lead and baryta water. Their presence in the air does not render it particularly dangerous, nor can they possibly give rise to specific diseases. The danger from the admission of sewer air into our houses consists in the possibility of its conveying the germs of disease. If sewer air is present in a house, more or less odor is necessarily present. The inodorous and deadly "sewer gas" of which we hear so much is a myth.

Dr. Fell spoke of the general sewage of the city and of the lack of proper ventilation, as many sewers open below the surface of the water, and thought some action by this society would be proper to further an effort towards improving the present system.

Dr. Fowler related a case of diphtheria, giving its course and surroundings.

Dr. Bartlett thought that the impure air caused physical weakness, thereby making the system more accessible to disease, and that the cause of sewer gas in buildings was, that the structures being warm caused a natural draught upward from the sewers through them. He thought that in itself it was not so poisonous as generally supposed. He reported that measles were now more fatal than at any time during the past six years.

Dr. Howe thought the sewer gas question was not as to chemical base, but of its infection with lower forms of animal or vegetable life, which are often multiplied with great rapidity.

*Reports of Committees*—The President reported that the Executive Committee had arranged for the use of the rooms now occupied, and their action met with the unanimous approval of the society.

Dr. Cronyn moved that this Association adjourn to meet again the first Monday in September. Seconded by Dr. Howe.

Dr. Rochester related past experiences, when adjournment was made for two months, and said it was difficult to get the members together again. Motion was lost.

Dr. Hopkins' motion was then put that when the Association adjourn, it shall be to the first Monday in July. Carried.

Dr. Brecht, the Treasurer, asked for instructions as to his duties regarding members who were more than five years in arrears for dues. Dr. Cronyn thought that two years should be long enough to wait for delinquent members and that decisive action should be taken to expel those who were so charged. After some discussion charges were preferred against several members, and the President referred the matter to a committee, consisting of Drs. Barrett, Bartow, Brecht, Mynter and Hopkins.

Dr. Fell invited all the members to be present at the meeting of the American Microscopical Society in Elmira in August next. Adjourned.

